# The effect of TEAS on the quality of early recovery in patients undergoing gynecological laparoscopic surgery: a prospective, randomized, placebo-controlled trial

**DOI:** 10.1186/s13063-019-3892-4

**Published:** 2020-01-08

**Authors:** Xiangdi Yu, Fangxiang Zhang, Bingning Chen

**Affiliations:** Department of Anesthesiology, Guizhou Provincial People’s Hospital, No. 83 Zhongshan Road Nanming district, Guiyang City, Guizhou Province China

**Keywords:** Transcutaneous electric acupoint stimulation (TEAS), Gynecological laparoscopic surgery, QoR-40, MMSE, VAS

## Abstract

**Introduction:**

In current study we assessed the effect of transcutaneous electrical acupoint stimulation (TEAS) on the quality of early recovery in patients undergoing gynecological laparoscopic surgery.

**Methods:**

Sixty patients undergoing gynecological laparoscopic surgery were randomly assigned to TEAS (TEAS group) or control group (Con group). TEAS consisted of 30 min of stimulation (12–15 mA, 2/100 Hz) at the acupoints of Baihui (GV20), Yingtang (EX-HN-3), Zusanli (ST36) and Neiguan (PC6) before anesthesia. The patients in the Con group had the electrodes applied, but received no stimulation. Quality of recovery was assessed using a 40-item questionnaire as a measure of quality of recovery (QoR-40; maximum score 200) scoring system performed on preoperative day 1 (T0), postoperative day 1 (T1) and postoperative day 2 (T2); 100-mm visual analogue scale (VAS) scores at rest, mini-mental state examination (MMSE) scores, the incidence of nausea and vomiting, postoperative pain medications, and antiemetics were also recorded. Results: QoR-40 and MMSE scores of T0 showed no difference between two groups (QoR-40: 197.50 ± 2.57 vs. 195.83 ± 5.17), (MMSE: 26.83 ± 2.74 vs. 27.53 ± 2.88). Compared with the Con group, QoR-40 and MMSE scores of T1 and T2 were higher in the TEAS group (*P* < 0.05) (QoR-40: T1, 166.07 ± 8.44 vs. 175.33 ± 9.66; T2, 187.73 ± 5.47 vs. 191.40 ± 5.74), (MMSE: T1, 24.60 ± 2.35 vs. 26.10 ± 2.78; T2, 26.53 ± 2.94 vs. 27.83 ± 2.73). VAS scores of T1 and T2 were lower (*P* < 0.05) in the TEAS group (T1, 4.73 ± 1.53 vs. 3.70 ± 1.41; T2, 2.30 ± 0.95 vs. 1.83 ± 0.88); the incidence of postoperative nausea and vomiting (PONV), remedial antiemetics and remedial analgesia was lower in the TEAS group (*P* < 0.05) (PONV: 56.7% vs. 23.3%; incidence of remedial antiemetics: 53.3% vs. 23.3%; incidence of remedial analgesia: 80% vs. 43.3%).

**Conclusion:**

The use of TEAS significantly promoted the quality of early recovery, improved MMSE scores and reduced the incidence of pain, nausea and vomiting in patients undergoing gynecological laparoscopic surgery.

**Trial registration:**

ClinicalTrials.gov, NCT02619578. Registered on 2 December 2015.

Trial registry name: https://clinicaltrials.gov

## Introduction

During the past four decades, gynecologic laparoscopy has evolved from a limited method to an advanced operative approach that frequently serves as a substitute for laparotomy. The advantages of laparoscopy over laparotomy include less postoperative pain, shorter hospital stays, and reduced blood loss [1–3]. However, during surgery CO_2_ increases the intra-abdominal and intrathoracic pressure, which leads to cardiac output decrease and increase in sympathetic activity in a reflex. On the other hand, CO_2_ accumulation in the body leads to hypercapnia, which indirectly stimulates aortic body chemosensory organs and carotid sinus, increasing the concentration of plasma catecholamines, cortisol and vasopressin [4, 5], these responses have an important impact on patient recovery after surgery.

Acupuncture is an ancient Chinese method to treat diseases and relieve pain. Transcutaneous electrical acupoint stimulation (TEAS), a noninvasive adjunctive intervention based on acupuncture, has been widely accepted and used worldwide [6]. To date, multiple studies have demonstrated that TEAS could reduce the use of intra-operative opioid drugs and the incidence of postoperative nausea and vomiting (PONV), and improve postoperative cognitive function [7, 8]. However, whether TEAS could improve the quality of early recovery after gynecologic laparoscopy is unknown. In this study we therefore investigated the effects of TEAS at the acupoints of Baihui (GV20), Yingtang (EX-HN3), Zusanli (ST36) and Neiguan (PC6) on the quality of early recovery in patients undergoing gynecological laparoscopic surgery.

## Methods

This is a double-blind randomized controlled trial (RCT). The study was conducted in accordance with the Declaration of Helsinki and was approved by the local Clinical Research Ethics Committee. Written informed consent was obtained from each participant. The study was approved by the Ethics Committee of Guizhou Provincial People’s Hospital.

### Patient population

Sixty patients undergoing elective gynecological laparoscopic surgery at Guizhou province people’s hospital with an American Society of Anesthiologists (ASA) classification physical status of I–II were recruited between November 2013 and November 2014; written consent was obtained from all patients. Exclusion criteria were recent use of TEAS or acupuncture, neural damage or infection along the meridian at which the acupoints lay, use of antiemetic in the previous week, regular use of opioids, hepatic dysfunction, confirmed renal impairment, diabetes mellitus, cognitive dysfunction and conversion to laparotomy during gynecologic laparoscopy.

### Randomization and blinding

**Fig. 1:**
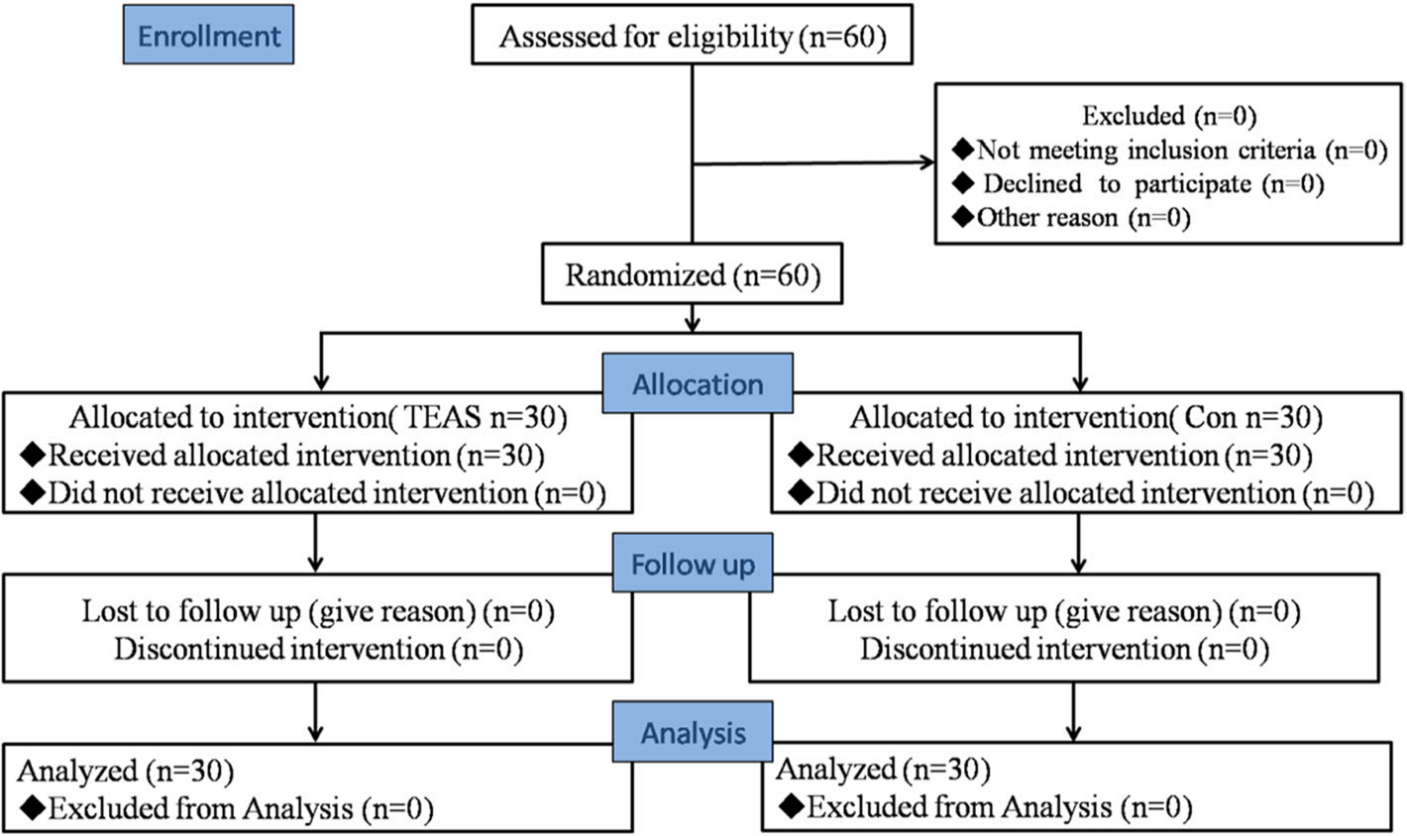
Consolidated standards of reporting trials (Consort) flowchart. TEAS, Transcutaneous electrical acupoint stimulation

Patients were assigned by the nurse to either TEAS stimulus (TEAS group) or control (Con group) on the basis of random numbers generated by a computer before the start of surgery (Fig. 1). Only the acupuncturist was informed by the nurse of the randomization allocation, just before the onset of TEAS. None of the anesthesiologists, surgeons, physicians in the post-anesthesia care unit (PACU), or participants were aware of the allocation. Blinding of the patients was ensured by using gel electrodes in the same therapeutic setting, which has previously been proved to be a successful strategy

[9].

### TEAS protocol

**Fig. 2:**
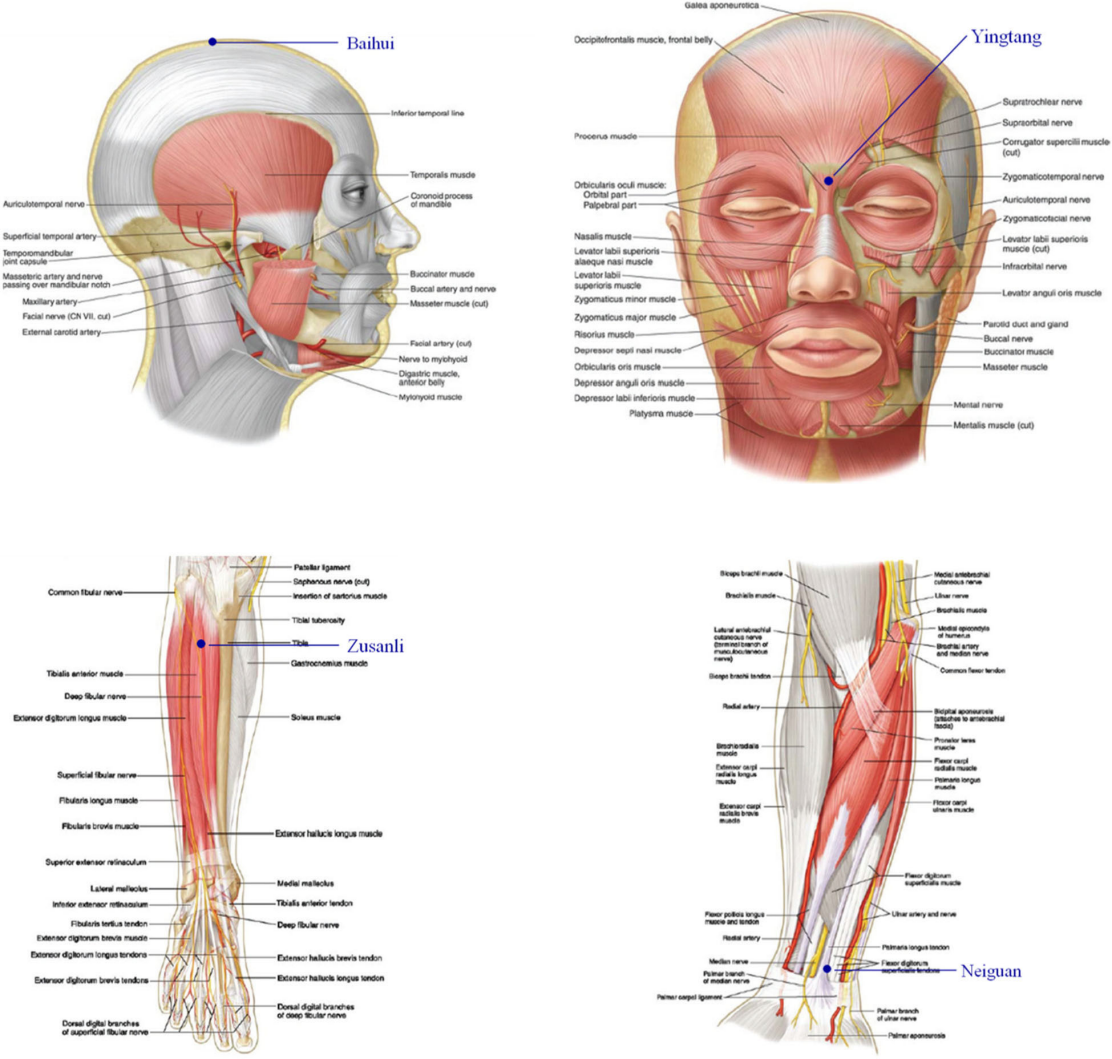
Location of acupoints. Baihui (GV20), located on the continuation of the line connecting the lowest and highest points of the ear, on the median line of the head, 7 cun above the posterior hairline, and 5 cun behind the anterior hairline; Yingtang (EX-HN-3), the middle point of the line between the brow bones; Zusanli (ST36), 3cun below Dubi (S35), one finger breadth from the anterior crest of the tibia; Neiguan (PC6), on the palmar side of the forearm and on the line connecting Quze (PC 3) and Daling (PC7), 2 cun above the crease of the wrist

An experienced acupuncturist performed TEAS for 30 min before anesthesia. According to the theory of traditional Chinese medicine, bilateral Baihui (GV20), Yingtang (EX-HN3), Zusanli (ST36) and Neiguan (PC6) were chosen as the acupuncture points. These acupoints were identified according to traditional anatomic localization (Fig. 2). Gel electrodes were applied to the skin after it had been cleaned with ethyl alcohol. The acupoints were then stimulated electrically with an intensity of 12–15 mA and dense-disperse frequency of 2/100 Hz for 30 min, using the Hwato electronic acupuncture treatment instrument (model SDZ-V, Suzhou Medical Appliances Co., Ltd., Suzhou, China). The intensity was adjusted to maintain slight twitching of local muscles according to individual maximum tolerance, indicating a satisfactory De-Qi phenomenon and thus adequate stimulation. The patients in the control group had the electrodes applied but received no stimulation.

### Anesthesia and perioperative management

One surgeon conducted all surgery according to a standard protocol; surgery commenced between 8:30 and 1:00 p.m. Anesthesia was induced intravenously (i.v.) with propofol and remifentanil using a target-controlled infusion (TCI) system. After loss of consciousness, vecuronium (0.1 mg·kg^− 1^) was administered i.v., and patients were orotracheally intubated 5 min later. Anesthesia was maintained with TCI of propofol and remifentanil. The depth of anesthesia was monitored using the bispectral index (BIS). Concentrations at the affected sites of propofol and remifentanil were adjusted to the hemodynamic index and BIS. Patients’ lungs were mechanically ventilated in a volume-controlled mode with a tidal volume of 8 ml·kg^− 1^ body weight during the operation. In both groups, remifentanil and propofol infusions were stopped 5 min before the end of surgery. Patients were extubated and transferred to the Post Anasthesia Care Unit (PACU) after surgery.

### End points

The primary end points were postoperative quality of recovery and cognitive functioning, and the secondary endpoint was anesthesia-related side effects, including pain scores, the incidence of nausea and vomiting and use of postoperative pain medications and antiemetics.

The Quality of Recovery-40 (QoR-40) is a validated scale with five domains [10–12]. These measure physical comfort, emotional state, physical independence, psychological support and pain. Each domain is scored to a maximum global score of 200. QoR-40 scores have been found to be associated with quality-of-life scales, patient satisfaction indices [13] and postoperative pain [14]. In the present study, QoR-40 evaluation was performed on preoperative day 1 (T0), postoperative day 1 (T1) and postoperative day 2 (T2).

The Mini-Mental State Examination (MMSE) is one of the most widely used assessment instruments of postoperative cognitive functioning, and screens domains of orientation to time and place, attention and memory, concentration, language and praxis [15]. Patient cognitive function was assessed using the MMSE on days T0, T1 and T2.

The visual analog scale (VAS) is widely used in behavioral science, and previous studies have reported their usefulness and validity [16, 17]. We used VAS scores to determine participants’ levels of pain at rest on postoperative day 1 (T1) and postoperative day 2 (T2).

### Data collection

Baseline data included demographics, body mass index (BMI) and ASA physical status. Surgical information recorded included anesthesia duration, surgery duration, estimated blood loss and all other intraoperative medications. BN, who was blinded to grouping, conducted the data collection and administered the questionnaires; FZ analyzed the data.

### Sample size

Sample size calculation was based on the primary outcome of QoR-40 scores and MMSE. We calculated that 16 patients in each group were required to detect a 30% difference between the groups, assuming two-sided type I error (α) of 0.05 and power of 80%. To account for potential loss to follow up and enable greater statistical power for secondary analysis, the sample size was increased to 60 patients (30 per group).

### Statistical analysis

All statistical analysis was based on the intention-to-treat principle. Normally distributed numeric data were analyzed using the *t* test and are reported as mean and standard deviation (SD). Variables measured at multiple time points were analyzed using repeated-measures analysis of variance. Dichotomous variables were presented as the number of patients (percent) and analyzed using the chi-square (*X*^2^) test and are reported as counts with percentages. Statistical significance was assessed at *P* < 0.05.

## Results

### Patient characteristics

**Fig. 3:**
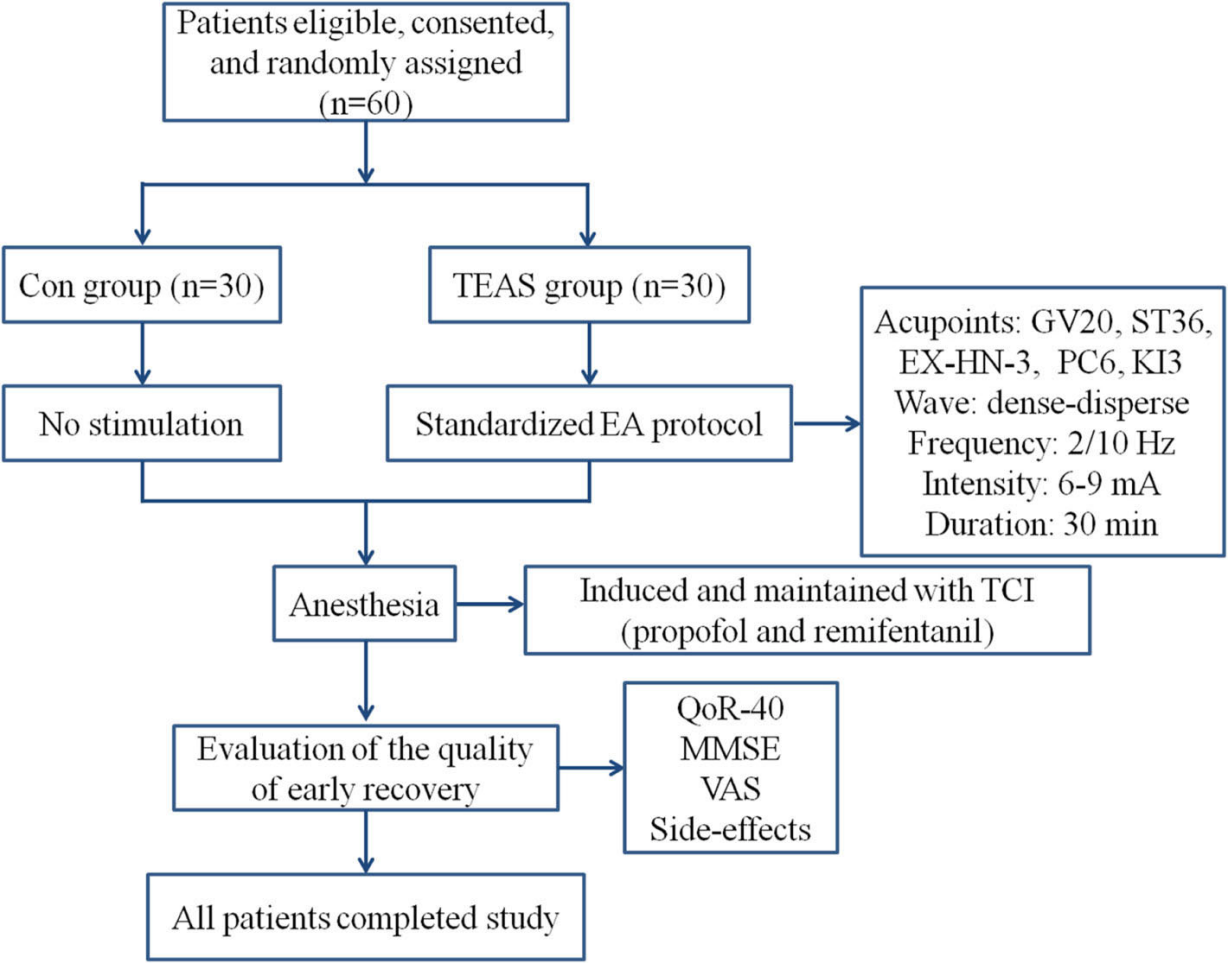
Flow diagram showing the study procedures and number of patients. TEAS, transcutaneous electric acupoint stimulation; TCI, target controlled infusion; EA, electroacupuncture; Con, control; MMSE, Mini Mental State Examination; VAS, visual analogue scale

Complete datasets were collected for all 60 participants and all the data were analyzed (Fig. 3). The characteristics of patients such as age, height, BMI, ASA physical status, anesthesia duration, surgery duration, estimated blood loss and fluid balance did not differ between the groups (Table 1).

**Table 1:** Patient characteristic and clinical characteristics of participants

	Contol group (*n* = 30)	TEAS group (*n* = 30)
Age, years	45.9 (17.5)	48.5 (16.2)
Height (cm)	157.9 (5.2)	158.3 (6.7)
Body mass index	22.1 (4.5)	22.0 (3.4)
ASA class		
I	23	22
II	7	8
Anesthesia duration (min)	190.3 (65.0)	224.2 (88.0)
Operation duration (min)	125.5 (62.1)	150.6 (76.4)
Intra-operative fluids (ml)	1710.6 (533.6)	1755.8 (479.3)

Data are presented as mean (SD). *TEAS* transcutaneous electrical acupoint stimulation, *ASA* American Society of Anesthesiologists

### Scores on the QoR-40 and MMSE (primary outcomes)

**Fig. 4:**
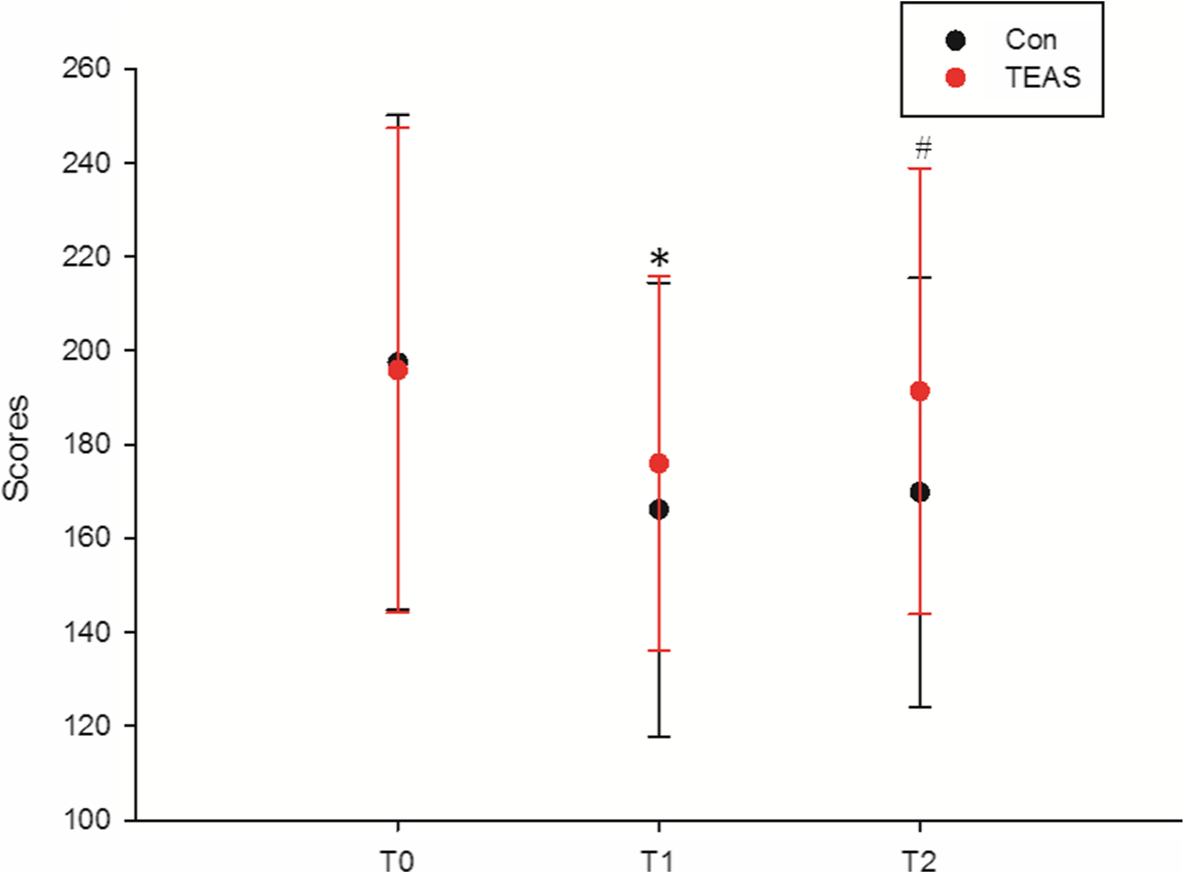
Scores on the global 40-item questionnaire as a measure of quality of recovery (QoR-40). When T0 was used as a covariate for a repeated measures model of T1 and T2, the significance of the interaction of group and time course was 0.003 and 0.036, respectively; *P = 0.040 control (Con) vs. electric acupoint stimulation (TEAS) at postoperative day 1 (T1); #*P* = 0.015 Con. vs. TEAS at postoperative day 2 (T2)

When evaluating the QoR-40, the global scores at baseline (T0) were similar. Following the procedure, global scores at T1 and T2 were significantly higher in the TEAS group (*P* = 0.040 and *P* = 0.015 for global QoR-40 at T1 and T2, respectively); when T0 was used as a covariate for a repeated measures model of T1 and T2, the significances for the interaction of group and time course were 0.003 and 0.036, respectively, indicating better quality of recovery with TEAS treatment (Fig. 4).

**Fig. 5:**
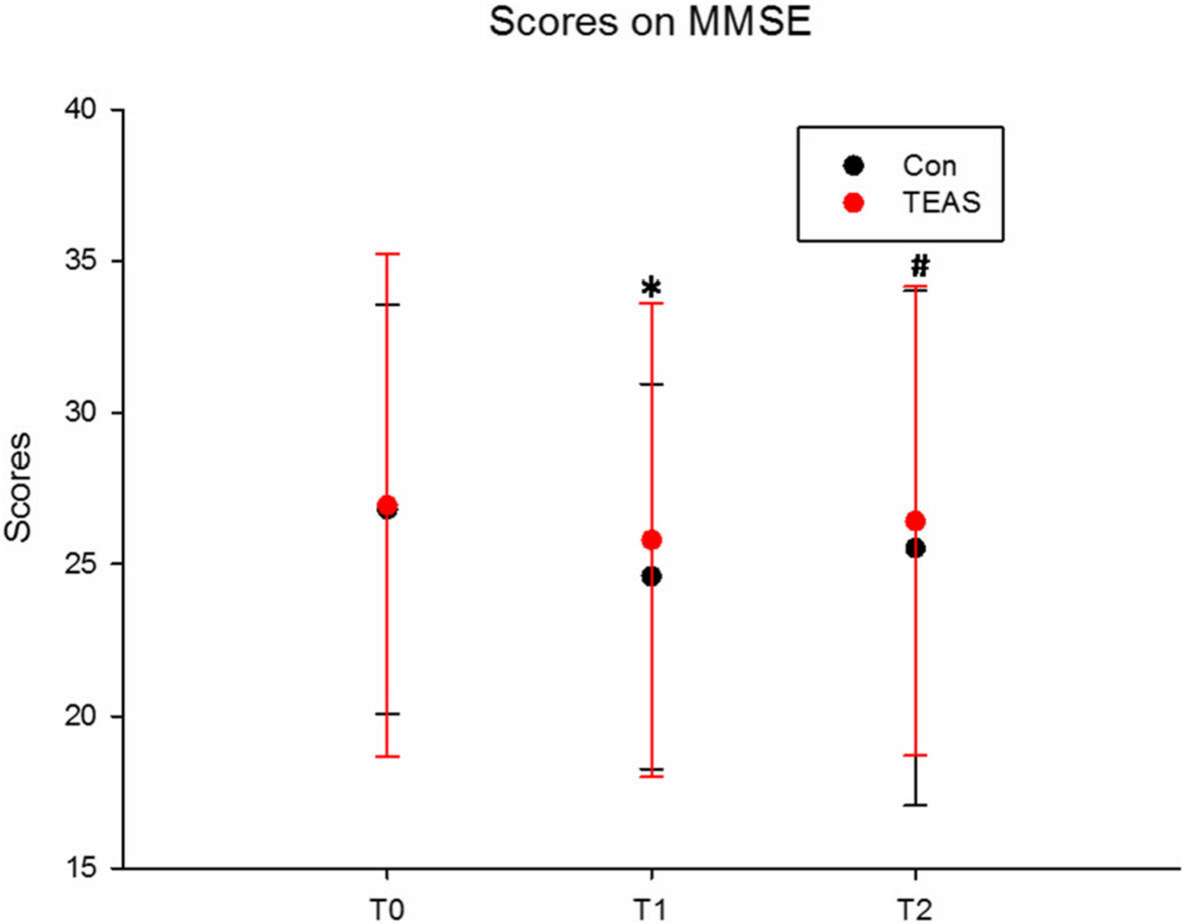
Scores on the Mini Mental State Examination (MMSE)

There were no significant differences in the baseline (T0) MMSE score for cognitive functioning in the two groups; however, the scores were significantly lower in the TEAS group compared with the Con group (*P* = 0.048 and *P* = 0.02 for T1 and T2, respectively); when T0 was used as a covariate for a repeated measures model of T1 and T2, the significances for the interaction of group and time course were 0.007 and 0.046, respectively (Fig. 5).

### Pain assessment and the incidence of nausea and vomiting, postoperative pain medications and antiemetics (secondary outcomes)

The VAS scores for pain at rest were significantly lower in the TEAS group compared with the Con group (*P* = 0.042 and *P* = 0.26 for T1 and T2, respectively) (Table 2). The frequency of nausea and vomiting within the first 24 h after surgery is shown in Table 3: 7 patients in the TEAS group and 17 patients in the Con group complained of nausea or vomiting with the first 24 h after surgery; 7 patients in the TEAS group and 16 patients in the Con group required tropisetron as antiemetic rescue therapy; 13 patients in the TEAS group and 24 patients in the Con group required sufentanil as analgesic rescue therapy (Table 3).

**Table 2:** Scores on the VAS

	Group	T0	T1	T2
VAS scores	Con	–	4.73 (1.53)	2.30 (0.95)
	TEAS	–	3.70 (1.53)*	1.83 (0.98)*

Data are presented as mean (SD). *VAS* visual analogue scale, *T0* baseline, *T1* postoperative day 1, *T2* postoperative day 2, *TEAS* transcutaneous electrical acupoint stimulation

*P < 0.05 *vs.* control group

**Table 3:** Incidence of nausea and vomiting, postoperative pain medications and antiemetics

Group	Cases of remedial analgesia	Cases of remedial antiemetic	PONV (%)
Con	24 (80%)	16 (53.3%)	17 (56.7%)
TEAS	13*(43.3%)	7 *(23.3%)	7* (23.3%)
*P* value	*P* < 0.01	*P* < 0.02	*P* < 0.01

Nausea was defined as a subjective unpleasant sensation associated with awareness of the urge to vomit; vomiting was defined as the forceful expulsion of gastric contents from the mouth brought about by the powerful sustained contraction of abdominal muscles

*PONV* Postoperative nausea and vomiting, *Con* control group, *TEAS* transcutaneous electrical acupoint stimulation

*P < 0.05 *vs.* control group

## Discussion

In this blinded RCT, use of TEAS at the acupoints of Baihui (GV20), Yingtang (EX-HN3), Zusanli (ST36) and Neiguan (PC6) for 30 min before anesthesia in patients undergoing gynecological laparoscopic surgery improved QoR and MMSE scores, reduced VAS scores and the need for pain medications and resulted in less postoperative nausea, vomiting and need of antiemetics. Our findings demonstrated that TEAS could promote patients’ recovery after laparoscopic surgery.

Laparoscopic surgery, with the advantages of less trauma and rapid recovery, is a method that is better accepted by patients. However, CO_2_ absorption and elevation of intra-abdominal pressure (IAP) from artificial pneumoperitoneum may impact patients’ postoperative recovery [18]. Previous studies have reported that the rates of postoperative symptoms in patients undergoing laparoscopic surgery were approximately 45% for pain, 17% for nausea, 8% for vomiting, 17% for headaches, 42% for drowsiness, 18% for dizziness, and 21% for fatigue respectively [19]. Therefore it is important to identify an easy and effective method to improve patient recovery after laparoscopic surgery.

Acupuncture has been used in China to treat a wide variety of diseases for thousands of years. Its major advantages are that it is minimally invasive, safe for high-risk patients and provides effective relief with a low risk of complications [20]. TEAS is the combination of traditional Chinese acupuncture and modern electrical techniques. An increasing number of clinical trials indicate that acupuncture and TEAS may be effective in reducing perioperative analgesic requirements, postoperative pain [21], and PONV [7]. Increasing evidence from animal experiments has shown that TEAS can attenuate cognitive deficits by means of maintaining sympathetic-parasympathetic balance, inhibiting apoptosis [22] and neuroinflammation [23] and regulating cell proliferation [24] in the hippocampus. Furthermore, TEAS at Zusanli (ST36) has been shown to accelerate the recovery of gastrointestinal motility after colorectal surgery [25].

In the present study, we found that TEAS at Baihui (GV20), Yingtang (EX-HN3), Zusanli (ST36) and Neiguan (PC6) for 30 min before anesthesia could promote the quality of early recovery, improve cognitive function, ameliorate postoperative pain and reduce the incidence of nausea and vomiting in patients undergoing gynecological laparoscopic surgery. The possible mechanisms were described as follows.

First, evidence from previous studies has shown that acupuncture and electroacupuncture (EA) performed at Baihui (GV20), Zusanli (ST36) and Yingtang (EX-HN-3) could regulate the function of the hypothalamic-pituitary-adrenal (HPA) axis [26] and antagonize the hyper-function of the HPA axis [27], possibly by regulating the relative enzymes (protein kinase A and protein kinase C in the experiment) in the signaling pathway in hippocampal cells. Evidence from animal experiments demonstrates that EA could significantly reduce the content of corticotrophin releasing factor (CRF) in the hypothalamus, and also reduce cortisol in the adrenal gland and plasma in depressed rats [28]. The HPA axis has many functions including regulation of appetite, sleep, sexual desires and adaptation to stress; dysfunction of the HPA axis is thought to be primarily responsible for psychological/behavioral symptoms (pain sensitivity, depression, fatigue) [29].

Second, it is generally agreed that acupuncture and EA can attenuate cognitive deficits by means of inhibiting the neuronic peroxidatic reaction with the hippocampal tissue and attenuating the inflammation in the central and peripheral nervous systems [30], and one of the major pathways is the cholinergic anti-inflammatory pathway (CAP), which depends on the α7 subunit of the acetylcholine receptor (AChR) on macrophages and other cells [31]. It was demonstrated that EA treatment could activate the CAP by stimulating the vagus nerve for which the major neurotransmitter is acetylcholine. Previous reports showed that vagus nerve stimulation could inhibit the production of pro-inflammatory cytokines [32]. We speculated that TEAS might be an effective method to improve postoperative cognitive function.

Third, an increasing number of clinical trials indicate that acupuncture and EA may be effective in reducing perioperative analgesic requirements and postoperative pain [21]. Studies demonstrate that acupuncture and EA could produce endogenous opioid peptides and stimulate endogenous descending inhibitory pathways. Analysis of cerebrospinal fluid from patients receiving acupuncture treatment reveals elevated levels of serotonin, endorphins and enkephalin [33]. Previous animal and human studies indicate that low-frequency (2 Hz) and high-frequency (100 Hz) EA induces induces differences in the release of a variety of opioid peptides. Studies have shown that TEAS at 2 Hz induces release of enkephlins and endorphine, TEAS at 100 Hz induces release of dynorphine [34–36] and only TEAS at 2/100 Hz induces release of all opioid peptides, resulting in their synergistic interaction [37]. This may explain our observation that TEAS could ameliorate postoperative pain and reduce analgesic rescue therapy in patients undergoing gynecological laparoscopic surgery.

Fourth, stimulation of the acupoint Neiguan (PC6) on the pericardium meridian has been used for centuries in China to treat morning and travel sickness and has been reported to be effective in preventing postoperative nausea and vomiting [38, 39]. The anti-PONV effects of acupuncture may be attributed to changes in the activity of neurochemicals, including endorphins, serotonin and norepinephrine in the central nervous system, which desensitize the “vomiting center” in the brain, ultimately strengthen the intrinsic anti-vomiting pathway; once the vomiting center in the brain is sensitized, it is difficult to desensitize it [40]. We assume that TEAS initiates release of more neurochemicals at an early stage and immediately desensitizes the vomiting center.

However, there were some limitations of this study. First, the sample size of 60 was small and comprised patients classified as ASA I and ASA II; a more diverse sample may have provided greater power to detect the effect of TEAS on the quality of early recovery in patients undergoing gynecological laparoscopic surgery. Second, the short study duration only permitted collection of limited data, which led to an underpowered trial. Third, due to economic restrictions we did not plan external monitoring and auditing. Thus, we cannot prove that our data really exits or that it is free from errors. However, for the sake of transparency, we state this limitation and we publish our results to the best of our understanding. Of course, before considering those results as definitive, they should be reproduced in a new trial with the highest standards for planning, monitoring, auditing and reporting.

## Conclusion

In conclusion, we found that TEAS significantly promoted the quality of early recovery, improved cognitive function, ameliorated postoperative pain and reduced the incidence of nausea and vomiting within the first 24 of surgery. In future studies, different acupoints could be used for different types of surgery on other parts of the body. We also plan to investigate the underlying mechanisms of TEAS-mediated therapy, ultimately to broaden the clinical appeal of the technique.

## Data Availability

Supporting data are available at clinicaltrials.gov (NCT02619578).

## Abbreviations

AP: Acupuncture
ASA: American Society of Anesthesiologists
BIS: Bispectral index
BMI: Body mass index
CAP: Cholinergic anti-inflammatory pathway
EA: Electroacupuncture
HPA: Hypothalamic-pituitary-adrenal
i.v.: Intravenously
MMSE: Mini-mental state examination
PACU: Post-anesthesia care unit
PONV: Postoperative nausea and vomiting
QoR-40: 40-item questionnaire as a measure of quality of recovery
RCT: Randomized controlled trial
TCI: Target-controlled infusion
TEAS: Transcutaneous electrical acupoint stimulation
VAS: Visual analogue scale
